# Advisability of Recording the Models of the Jaws of Aviators

**Published:** 1919

**Authors:** 


### Advisability of Recording the Models of the Jaws of Aviators.

By Lieut.-Col. William A. Squires, D. C. *Journal Association Military Dental Surgeons of U. S.*, October, 1918.

On account of the large number of fractures of the jaws in aviators who fall and are not killed, Lieut.-Col. Squires suggests that good impressions in modeling compound should be made of the upper and lower jaws of every aviator in the Service, and from these impressions hard plaster or metal models made. These models should be as thin as possible, and should be sent to each aviator's station with his service record. With the aid of these models, in case of fracture of the jaw, the dental surgeon can replace the teeth and jaws in their normal position and decide upon the splint to be used, without waiting for the man to recover from the shock of the accident sufficiently to make it possible to take an impression of the jaw.

